# Reliability and Reproducibility of Absolute Myocardial Blood Flow: Does It Depend on the PET/CT Technology, the Vasodilator, and/or the Software?

**DOI:** 10.1007/s11886-021-01449-8

**Published:** 2021-01-22

**Authors:** K. Lance Gould, Linh Bui, Danai Kitkungvan, Monica B. Patel

**Affiliations:** 1 Weatherhead PET Center For Preventing and Reversing Atherosclerosis, Division of Cardiology, Department of Medicine, McGovern Medial Medical School, University of Texas, and Memorial Hermann Hospital, Houston, TX USA; 2 Weatherhead PET Center For Preventing and Reversing Atherosclerosis, McGovern Medical School, University of Texas Health Science Center at Houston, 6431 Fannin St., Room MSB 4.256, Houston, TX 77030 USA; 3 Division of Cardiology, McGovern Medical School, Houston, TX USA

**Keywords:** Coronary artery disease, Revascularization, Myocardial perfusion, Coronary flow reserve, Coronary flow capacity, Cardiac positron emission tomography

## Abstract

**Purpose of Review:**

The COURAGE and ISCHEMIA trials showed no reduced mortality after revascularization compared to medical treatment. Is this lack of benefit due to revascularization having no benefit regardless of CAD severity or to suboptimal patient selection due to non-quantitative cardiac imaging?

**Recent Findings:**

Comprehensive, integrated, myocardial perfusion quantified by regional pixel distribution of coronary flow capacity (CFC) is the final common expression of objective CAD severity for which revascularization reduces mortality. Current lack of revascularization benefit derives from narrow thinking focused on measuring one isolated aspect of coronary characteristics, such as angiogram stenosis, its fractional flow reserve (FFR), anatomic FFR simulations, relative stress imaging, absolute stress ml/min/g or coronary flow reserve (CFR) alone, or even more narrowly on global CFR or fixed regions of interest in assumed coronary artery distributions, or in arbitrary 17 segments on bull’s-eye displays, rather than regional pixel distribution of perfusion metrics as they actually are in an individual.

**Summary:**

Comprehensive integration of all quantitative perfusion metrics per regional pixel into coronary flow capacity guides artery-specific interventions for reduced mortality in non-acute CAD but requires addressing the methodologic questions in the title.

## Introduction: Convenience Methodology or Clinical Coronary Physiology?

The assigned title questions focus on methodology that requires reviewing several broader issues as the basis for technical, data-driven answers.

Cardiology thinking in non-acute CAD is dominated by methodology for measuring one isolated aspect of coronary anatomy or physiology, such as angiogram stenosis, its fractional flow reserve (FFR), anatomic FFR simulations, relative stress imaging, or coronary flow reserve (CFR). For even the few centers quantifying myocardial perfusion, this narrow methodology orientation focuses simplistically on coronary flow reserve (CFR) or stress perfusion alone or more narrowly on global CFR in assumed fixed regions of interest of the three major coronary artery distributions or in assumed 17 arbitrary bull’s-eye segments in which perfusion is measured as opposed to actual arterial distributions as they are in an individual.

Faced with complexity, cardiology focuses on a simplifying single measurement rather than a simple, easily understood comprehensive integrated display of complex physiologic data determining outcomes. Nature’s integrated coronary physiology [1, 2, 3•, 4•, 5•] evolving for survival over millions of years does not correspond to or care about narrow views of a single measurement by any methodology. Since quantifying myocardial perfusion is essential for guiding interventions [1, 2, 3•, 4•, 5•, 6••, 7••, 8], this overview summarizes a data-driven definite hierarchy of clinical relevance or value for global and regional stress ml/min/g, CFR, and their combination per pixel as coronary flow capacity (CFC) from outcomes backwards to specific technology relevant to those outcomes for answering the title questions.

## Start with Outcomes, Work Backwards to Technology

Therefore, answers to the title questions start with clinical purpose, reporting, and outcomes of comprehensive integrated absolute myocardial perfusion and working backwards to define the conceptual and technical requirements for achieving optimal outcomes. These requirements are based on 50 years of the senior author developing invasive and non-invasive quantification of physiologic coronary function. This search began before cardiac PET existed, evolved through critical experimental and clinical analysis of stenosis on coronary angiograms and pressure-flow equations [2, 3•], to experimental and clinical pharmacologic stress imaging [8] to routine diagnostic quantitative myocardial perfusion by positron emission tomography (PET) [1, 2, 3•, 4•, 5•, 6••, 7••, 8] as the final common expression of upstream epicardial pressure-flow pathophysiology.

Understanding quantitative myocardial perfusion requires a sustained, objective, self-criticism of cardiac positron emission tomography (PET) for errors or systematic flaws compromising optimal patient outcomes. Coronary flow reserve (CFR) and fractional flow reserve (FFR) derived from these original pressure-flow equations are now common in cardiology with PET universally accepted as the reference standard for quantitative myocardial perfusion in textbooks [2, 3•].

However, both CFR and FFR are currently somewhat mis-viewed as stand-alone methodologic endpoints or gold standards rather than recognized as one limited facet of the larger integrated comprehensive coronary behavior determining patient outcomes. Moreover, in current practice, both are profoundly flawed by failure to integrate absolute rest-stress flow, CFR, and their combination as coronary flow capacity (CFC) per regional pixel quantifying size-severity of abnormalities in arterial distributions as they actually are. As currently commonly used, both also fail to identify or quantify widespread reduced subendocardial perfusion during stress, with or without angina, due to diffuse CAD with or without focal stenosis [5•] as the physiologic equivalent of widespread coronary atherosclerosis by IVUS or coronary calcium. Cardiac PET as currently commonly used also has a number of technical but fixable limitations addressed here in response to the title questions.

To achieve optimal clinical care, this critical testing and continuous revisions of cardiac PET in this lab extend from hardware to software to acquisition protocols to pharmacologic stress to clinical displays. Importantly, it includes specific reporting text recommendations toward or away from invasive procedures of specific arteries based on integrated, comprehensive interpretation of myocardial perfusion based on outcomes that invasive cardiologists here request and expect interfaced with clinical judgment and patient preference.

## What Does PET That Determines Outcomes Look Like?

**Fig. 1:**
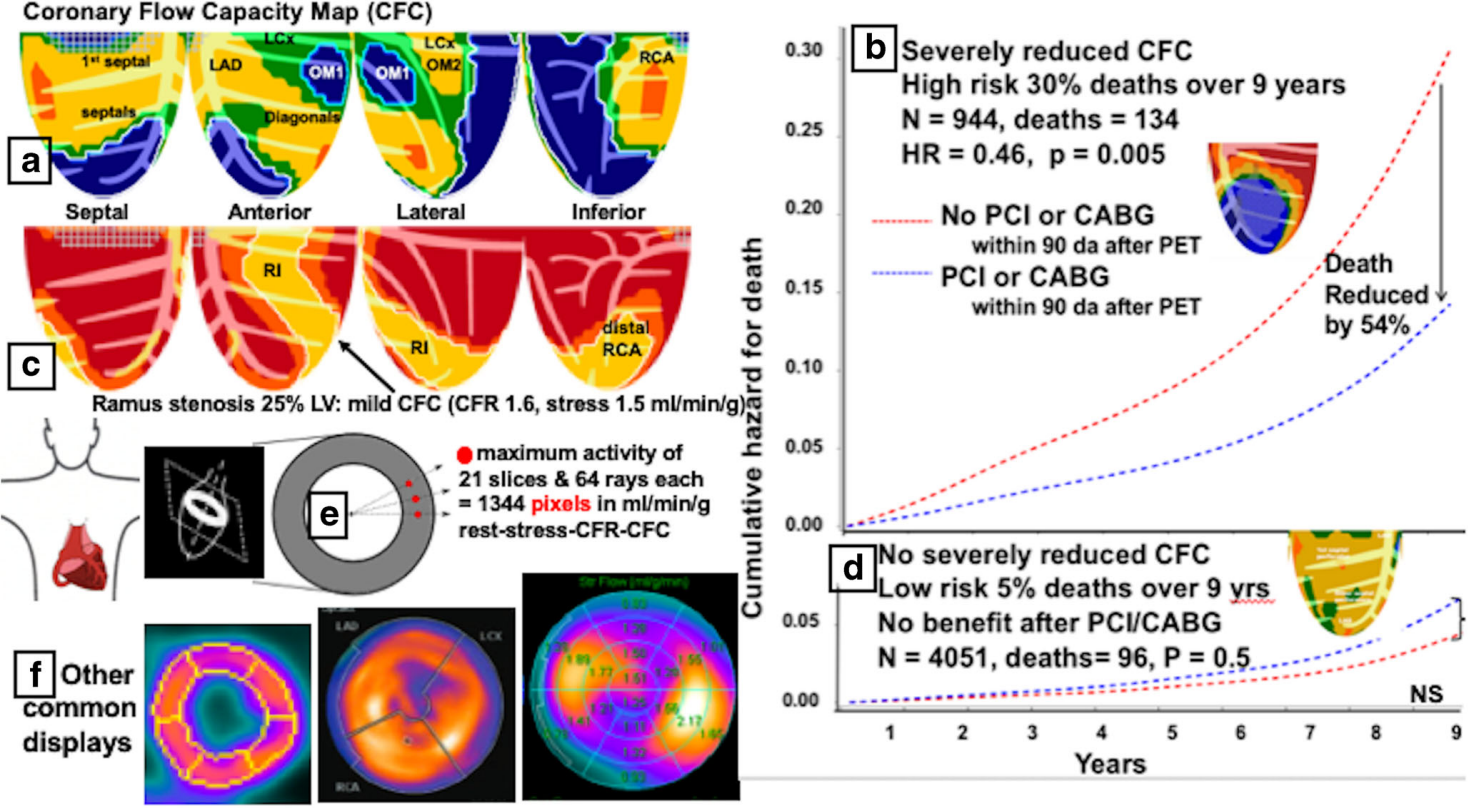
Summary figure. **a** Complex CAD. **b** All-cause mortality for severely reduced CFC (blue) with and without revascularization. **c** Mildly reduced CFC (yellow) in distribution of a ramus intermedius branch. **d** All-cause mortality for mildly or moderately reduced CFC (no blue) with and without revascularization. **e** Radial sampling on tomographic slices for determining quantitative myocardial perfusion in ml/min/g. **f** Standard arbitrary regions of interest in which ml/min/g are measured instead of actual arterial distribution for each individual

In Fig. 1a, coronary flow capacity (CFC) maps complex diffuse CAD (yellow) with severe stress abnormalities (blue) comprising 38% of the left ventricle (LV) indicating subtotal or occlusions of distal LAD, a small OM1, distal LCx, and distal RCA with myocardial steal indicating collaterals to viable myocardium [4•]. The CFC green regions are border zones of moderately reduced transmural perfusion around severe transmural ischemia (blue). The white line outlines cumulative size and severity of abnormalities. The light blue line circumscribes the size-severity of the small separate OM1 defect as 4% of LV with severely reduced CFC (blue) and CFR 0.6 (steal) and stress 0.7 m/min/g (ischemic level) also characterizing the larger abnormality.

The PET report detailed these artery-specific abnormalities as above and concluded: “Depending on clinical judgement and patient preferences, coronary angiogram is a valid option but will likely reveal diffuse CAD, distal subtotal or occlusion of the three major coronary arteries with complex anatomy suboptimal for revascularization hence favoring medical treatment.” Angiogram confirmed exactly these PET results with a decision for medical treatment.

The technical basis for precisely mapping comprehensive, integrated, myocardial perfusion for guiding interventions derives from pixel determinations of both CFR and stress ml/min/g in arterial distributions as they actually are in individuals. Various combinations of CFR and stress ml/min/g for each of 1344 pixels in an LV image comprise an enormous volume of data that need simplifying while still retaining physiologic accuracy and clinical relevance [1, 2, 3•, 4•, 5•, 6••, 7••].

Accordingly, each CFC pixel combination of CFR and stress ml/min/g is color-coded for 5 well-defined clinical groups and back-projected to its spatial position in the left ventricular image with percent of LV for each CFC severity range as follows: **red**, normal, defined from 125 healthy, young volunteers with no risk factors (CFR > 2.9 and stress perfusion > 2.17 cc/min/g); **orange**, minimally reduced, defined by risk factors only with no clinically manifest CAD (CFR > 2.38 to 2.9 and stress perfusion > 1.82 to 2.17); **yellow**, mildly reduced, defined by documented stable CAD without angina or ST depression on ECG during dipyridamole stress (CFR > 1.6 to 2.38 and stress perfusion > 1.09 to 1.82); **green**, moderately reduced, with possible ischemia defined by angina or ST depression ≥1 mm with a relative stress defect (CFR > 1.27 to 1.6 and stress perfusion > 0.83 to 1.09); **blue**, severely reduced with definite ischemia defined by angina and ST depression ≥ 1 mm and a relative stress defect (CFR 1.0 to 1.27 and stress perfusion ≤ 0.83); **dark blue**, defined by myocardial steal with stress perfusion falling below rest perfusion (CFR < 1.0) [1, 2, 3•, 4•, 5•, 6••, 7••].

Patients referred for PET at this center have high prevalence of known CAD, risk factors, or symptoms; 20.4% had severely reduced CFC (blue > 0) with rare or essentially no false positives due to compulsively co-registering emission and transmission data in every patient. In addition, for questionable inferior apical motion artifacts on whole cycle images, perfusion on systolic images is measured that removes motion artifact and minimizes partial volume loss for the thickest systolic LV wall. False negatives for significant CAD are also rare as evidenced by PET-guided angiograms that show significant CAD with 82% having revascularization procedures and the remainder having diffuse or complex CAD not suitable for revascularization as in Fig. 1a. A rare patient may have non-flow-limiting stenosis by dipyridamole PET but exercise spasm of treadmill by relative images using N-13 ammonia. The size-severity of CFC abnormalities predicts risk of adverse coronary events and their change with and without revascularization shown in Fig. 1b.

## Outcomes for Severe Coronary Flow Capacity

Severely reduced CFC (blue) shown in Fig. 1b associates with high mortality that is significantly reduced by 54% after revascularization in a non-randomized prospective database [6••, 7••], now comprising over 8000 routine clinical PETs with follow-up over 10 years. Regional or global CFR or stress perfusion alone separately of comparable severity to the CFC thresholds above is associated with significant but lower risk than CFC and no survival benefit after revascularization [6••, 7••].

However, others report global CFR associated with reduced mortality after revascularization [9•, 10]. While confirming the benefit of PET, those studies are difficult to interpret as a guide to invasive procedures for two reasons. First, global CFR fails to differentiate regional abnormalities due to flow-limiting stenosis from diffuse or complex CAD thereby precluding which PET abnormalities benefited. Second, quantitative perfusion data was reported as specifically withheld from cardiologists making revascularization decisions. The consequences of these two issues are addressed subsequently.

## Global vs Regional Perfusion

While widely reported [9•, 10], global CFR or stress perfusion alone, or perfusion within fixed regions of interest (ROIs) for assumed standard or angiogram defined arterial distributions, average the perfusion of different neighboring arterial distributions within each arbitrary ROI [1, 2, 3•, 4•, 5•, 6••, 7••, 11•, 12]. Such arbitrary ROIs preclude tracking severity of iso-contour boundaries for precise size and severity of artery-specific stenosis done in Fig. 1a and c. Based on pixel values of CFR and stress perfusion comprising CFC, the white or blue lines precisely circumscribe separate abnormalities in Fig. 1a quantified as % of LV with specific CFC severity, CFR, and stress ml/min/g for each separate specific region. The mildly reduced CFC (yellow) indicates diffuse CAD in addition to focal occlusions or flow-limiting stenosis (blue) that are essential for planning clinical management.

Therefore, neither stenosis nor diffuse CAD can be quantified by global perfusion that averages regional defects into a non-specific global mean of stress ml/min/g and CFR. In contrast, CFC by definition of its pixel values maps severity and size of artery-specific abnormalities as they actually are in an individual [1, 2, 3•, 4•, 5•, 6••, 7••, 8].

## Outcomes for Mildly Reduced Coronary Flow Capacity

The patients undergoing cardiac PET at this PET center have a high prevalence of CAD or risk factors with 75.2% having coronary calcification ≥ 120 Hounsfield units, 66.7% having abnormal PETs, and 20.4% with severely reduced CFC (blue). Of PET-directed angiograms, 82% underwent revascularization with outcomes shown in Fig. 1b [6••, 7••].

In contrast, for all PETs with non-severe CFC seen in Fig. 1c and d, risk of adverse events is low. For those having revascularization despite only mild or moderately abnormal CFC, mortality was not reduced but was insignificantly increased. Thus, CFC also eliminates unnecessary diagnostic angiograms not leading to revascularization. Moreover, in a larger cohort study of global CFR, revascularization for patients with CFR over 1.8 had significantly increased mortality [9•] due to risk of the procedure being greater than risk of physiologically mild CAD.

## Quantitative Perfusion per Pixel vs Arbitrary Regions of Interest

The technical basis for CFC maps defining precise size-severity of abnormalities in specific arterial distributions derives from color-coded pixel CFC values of stress ml/min/g and CFR. Maximum activity along 64 radii in each of 21 short-axis tomographic slices provides μCi/g of myocardium with maximal statistical certainty, as shown in Fig. 1e [1, 2, 3•, 4•, 5•, 6••, 7••, 11•, 12–14]. Pixel data allow size and severity of arterial distribution as it actually is for either severe abnormalities as seen in Fig. 1a or mild abnormalities as seen in Fig. 1c and d.

Arbitrary ROIs for assumed 3 artery distributions or the 17 bull’s-eye segments as shown in Fig. 1f do not allow such specific artery size-severity quantification. The bull’s-eye display in Fig. 1f did not resolve an interventional decision by the cardiologist who referred the patient for the CFC map in Fig. 1c showing mildly reduced CFC in the distribution of a ramus intermedius as the basis for his decision to treat medically with event-free follow-up to the present [11•].

As in the three displays in Fig. 1f, drawing endocardial boundaries or fixed regions of interest in which ml/min/g is averaged for overlapping arterial distributions distorts regional quantitative data. Moreover, arbitrary segmentation of epicardial-endocardial borders within which perfusion is averaged relies on boundary transitions of low count density and poorest statistical certainty that degrade certainty of size of regions in which perfusion is averaged [11•, 15].

## Simple Clinical Displays of Correct Complex Data to Guide Interventions

Standard bull’s-eye displays distort the visual view and quantitative data sufficiently to obscure essential regional information and interpretation for guiding interventions. The third case shown in Fig. 1f had the initial standard 17-segment PET display that failed to guide the referring cardiologist to medical or interventional management. Therefore, he was sent for PET with CFC that was immediately clear to the cardiologist as showing mild low-risk narrowing of the ramus intermedius shown in Fig. 1c leading to medical treatment [11•]. Clinical PET needs simple displays that correctly integrate complex data related to outcomes needed for optimal decisions by interventionalists unfamiliar with quantitative perfusion, its risks, or benefits with and without revascularization [5•, 6••, 7••, 11•].

## Test-Retest Reproducibility

Test-retest precision (coefficient of variance—COV) in the same patient was determined by serial quantitative imaging minutes and days to weeks apart in 100 healthy young volunteers with no risk factors and in 120 volunteer patients with risk factors or known CAD [11•, 12–15]. The test-retest COV for serial stress ml/min/g minutes apart is ± 10%. When serial images in the same patient are separated by days to weeks, the COV for stress perfusion is ± 20%, thereby indicating that half (10%) the variability is biological and the other half (10%) is methodologic variability.

The Kolmogorov-Smirnov statistic and CFC cumulative histogram distributions for these serial PETs were identical with extremely low KS statistic of 0.01. This statistic means that among separate serial CFC maps on different days in the same patient, the cumulative CFC histogram distribution differed by only 1% or less of LV. Thus, the variability of CFC regional distribution is much less than either stress ml/min/g or CFR.

A major reason for the greater variability of quantitative perfusion in literature is due to variation of ROI selection for arterial input activity [3•, 4•, 11•, 15]. Due to translational motion during cardiac and respiratory cycles, the left atrium (LA) commonly moves in and out of fixed ROIs located by back-projection from late myocardial images or from the AV ring on CT images. ROIs located on maximal left atrial activity on good images of LA activity acquired or summed over minutes provide the most reliable arterial input. Determining optimal LA ROI for arterial input on high-quality LA images has also been confirmed for MRI quantitative myocardial perfusion [16].

## Dipyridamole, Adenosine, Regadenoson, Dobutamine, Caffeine, and Exercise

Adenosine and dipyridamole produce comparable stress ml/min/g and CFR [17]. Radionuclide injection within 20 s after regadenoson injection produces stress perfusion that is 20% less than dipyridamole in the same patient [18]. If radionuclide is given at 55 s after regadenoson injection, stress perfusion is somewhat better at 10% less than dipyridamole. Therefore, in some patients, regadenoson fails to reveal true size and severity of regional defects or may cause appearance of diffusely reduced stress perfusion or CFR erroneously suggesting diffuse CAD. Alternatively, regadenoson is less stressful and shorter for fragile patients or those with borderline blood pressure or known severe CAD in whom identifying a regional source of angina may be important for limited artery-specific intervention on refractory angina.

Blood caffeine may significantly degrade diagnostic accuracy of stress PET for all three vasodilator stressors [19]. Measurable caffeine levels are reported in 20% of patients in the literature. By verbal and written emphasis with phone reminders 2 days before every scheduled PET, the prevalence of measurable blood caffeine can be reduced to 6% of subjects undergoing stress PET, comparable to the prevalence of genetic slow caffeine metabolizers [19]. Blood caffeine levels are checked in every patient in this lab with repeat PET done if caffeine compromised quantifying severity for interventional decisions. Treadmill exercise precludes quantitative myocardial perfusion since arterial input cannot be determined.

## Radionuclides for Quantitative Perfusion—Rb-82, N-13 Ammonia, and O-15 Water

Each radionuclide has their appropriate perfusion model validated compared to microspheres [20–23]. Claims of one being better than another based on its extraction fraction for Rb-82, N-13 ammonia, and F-18 flurpiridaz or lack thereof for O-15 water is a false claim reflecting ignorance or bias of the claimant who discounts their validation with their appropriate flow models. Each has separate strengths and weaknesses, and requires compulsive adherence to the model, the arterial ROIs, and all the technical factors above. Done properly, all are accurate for determining quantitative myocardial perfusion. For serial rest stress myocardial perfusion imaging, the long half-life of F-18 compromises quantification of the second of serial images due to residual activity.

## Subendocardial Perfusion and No-Stenosis Angina

As reported [5•], of 5900 routine quantitative PETs, 362 (6.1%) with no regional stress abnormalities or normal angiograms had definite to severe angina during dipyridamole stress. Most, 341/5900 or 5.8%, had mean transmural global CFR ≥ 2.2 indicating good microvascular function but reduced subendocardial perfusion on tomographic views due to diffuse epicardial CAD. Only 21/5900 or 0.4% had reduced CFR ≤ 2.2, indicating reduced microvascular function. Thus, 341/362 or 94.2% of no-stenosis angina is due to diffuse epicardial CAD not microvascular dysfunction (Table 1) [5•]. The normal mean transmural stress perfusion indicates excellent microvascular function producing high epicardial coronary artery blood flow through diffuse epicardial CAD that decreases perfusion pressure and thence reduced subendocardial perfusion during stress tachycardia despite no flow-limiting stenosis [2, 3•, 4•, 5•, 8].

**Table 1:** No-stenosis angina

No-stenosis angina	CFR ≤ 2.2	CFR > 2.2
Number (% of 5900)	21 (0.4%)	341 (5.8%)
Microvascular function	Impaired	Good
Female	62%	42%
Risk factors	Yes	Yes
MI or death over 9 years	9.5%	4.4%

For these not uncommon cases, the usual diagnosis of microvascular angina is simply wrong due to cardiologists failing to register or remember definitive experimental demonstration of reduced subendocardial perfusion during hyperemic flow through even mild focal or diffuse coronary narrowing [2, 3•, 4•, 5•, 8], now confirmed clinically by quantitative PET [5•].

## PET-CT Scanners—2D vs 3D

The HeartSee flow model (510K-171303) with a 2-min arterial phase image and 5-min myocardial image for Rb-82 and N-13 ammonia is validated experimentally [20, 21] and clinically as outlined above [1, 2, 3•, 4•, 5•, 6••, 7••, 11•, 12–15, 17–21] for full-dose Rb-82 or N-13 ammonia with the appropriate flow model for each and longer myocardial phase images for N-13 ammonia. Direct objective comparison to multi-compartmental models by another PET center concluded that the retention model of HeartSee flow software provided for Rb-82—“higher sensitivity for detection and localization of abnormal flow and myocardial perfusion reserve…without the computational complexity and sensitivity to noise – of the multi-compartmental model” [23].

However, 3D acquisition on BGO 2D-3D scanners fails to acquire accurate arterial input over minutes-long single arterial phase images due to inadequate corrections for random coincidences, dead time, and scatter [11•, 12]. As a feasibility study for these BGO 2D-3D scanners, serial short 15-s images each corrected separately then summed for a 2-min arterial phase image provide adequate quality arterial phase images on which optimal ROI for arterial input can be determined [11•, 12]. The HeartSee flow model has been validated with the most current 3D solid-state PET-CT scanners acquiring list mode data with corrections before summing into the 2-min arterial phase for optimal arterial input and 5-min myocardial phase images for Rb-82, or longer myocardial phase for N-13 ammonia.

Any shifting of images for correct co-registration of emission and transmission data needs to be done after summing the corrected serial images. The reason is that the short serial 3D images are still so noisy that matching emission with transmission borders is subject to substantial error in some but not other images in the arterial series, thereby corrupting co-registration of the summed image. As the basis of the most accurate perfusion measurements, the summed images need correct co-registration of high-density definite borders before LA ROI selection.

## Comparison with the Literature

The answers to the title questions in this paper are addressed and referenced based on the methodology, protocols, interpretation, reporting, clinical application, and outcomes at this center since 2007 that is substantially different than most other PET facilities. Word limits preclude detailed comparison among diverse PET facilities. However, an overview of salient literature is appropriate for similarities and differences. Bober et al. [24•] reported that revascularization yielded significant improvements in stress perfusion in ml/min/g when targeted to regions with reduced coronary flow capacity (CFC) or relative perfusion abnormalities on baseline PET. When revascularization was performed in regions without reduced CFC, stress perfusion did not improve.

Patel et al. reported [9•, 25] a global CFR threshold at which revascularization reduced mortality. The (−)1 SD limits of this CFR threshold was 1.3 to 1.4 [Figures 3 and 5 of reference 9•], comparable to the CFR threshold for severely reduced CFC blue of 1.27 associated with high mortality risk reduced after revascularization [1, 2, 3•, 4•, 5•, 6••, 7••, 8]. However, global CFR does not delineate or quantify regional abnormalities due to artery-specific stenoses that are the targets of revascularization. In addition, these reports state specifically that the quantitative myocardial perfusion data was withheld from referring physicians (for 6 years on 12,594 patients) [9•, 25]. Withholding quantitative perfusion data from clinicians managing the patients would likely limit demonstrating any benefit of quantitative perfusion on patient management. Alternatively, withholding quantitative perfusion data may reflect not understanding its clinical use or due to sufficient measurement variability precluding interpretation for individual patients.

Taqueti et al. [10] reported risk stratification with reduced mortality after coronary artery bypass surgery (CABG) compared to no CABG for global CFR ≤ 1.5 but no mortality benefit after PCI. Gupta et al. [26] reported risk stratification for CFR alone as not improved by the addition of global stress perfusion in ml/min/g. However, for both studies, the global rest-stress perfusion and global CFR average out all regional rest-stress defects and all regional abnormal CFR defects. In contrast, coronary flow capacity is by definition the regional per-pixel distribution of stress perfusion and CFR specifically designed to quantify the common heterogeneous differences of these two metrics in arterial distributions as they actually are to guide management of CAD in an individual patient.

Danad et al. [27•] reported PET predicting FFR ≤ 0.8 as superior to CT angiogram with the strong conclusion that “This controlled clinical head-to-head comparative study revealed PET to exhibit the highest accuracy for diagnosis of myocardial ischemia. Furthermore, a combined anatomical and functional assessment does not add incremental diagnostic value but guides clinical decision-making in an unsalutary fashion”.

Driessen et al. [28] compared simulated FFR predicted by CTA analysis (FFRct) versus FFR measured directly by PET and both compared to pressure-derived FFR. Analysis of all subjects for intent to diagnose showed that PET was superior to FFRct for predicting pressure-derived FFR, as expected since pressure-derived FFR, a relative CFR, was originally validated by comparison with PET relative stress perfusion [29]. However, the authors then excluded the 17% of cases in which FFRct could not be determined due to inferior quality in order to claim a primary conclusion that FFRct was superior to PET for predicting pressure-derived FFR. This paper excluding FFRct failures is co-authored by founders of the commercial distributor of FFRct with a conclusion directly opposite to the correct intent-to-diagnose analysis including all subjects showing PET superior to FFRct. Its conclusion is also opposite to the prior paper strongly critical of CTA by the same academic authors in the Danad paper above without commercial co-authors.

Due to these conflicting issues in the literature, we have focused this review on the large PET database of approximately 9000 cases acquired at this institution since 2007 using the same protocols, scanner, radionuclide, the same software for automated quantification of all perfusion metrics by the same technologists, physicians, database, the same systematic interpretations, and cardiac PET imaging and consultation report with specific recommendations, “Depending on clinical judgement for medical treatment or for invasive procedures in specific arterial distributions”. Its advantages include consistent data accumulation, reproducibility, and systematic follow-up protocols approved by UT Committee for the Protection of Human Subjects. The limitations include single-center data and different diverse PET technologies used elsewhere.

## Conclusion: The Greatest Problem and a Solution

Answers to the three title questions are yes, yes, and yes. However, cardiology thinking needs to expand beyond fixation on current convenient or familiar methodologies at hand—angiograms, intracoronary FFR, coronary flow velocity, angiogram simulations of FFR, relative perfusion imaging, stress ml/min/g, or CFR alone, to understanding integrated comprehensive myocardial perfusion that determines mortality, expressed as coronary flow capacity as the core final expression of upstream pathology—toward optimal outcomes as illustrated in Fig. 1. This goal is achievable by attention to the technical details reviewed here for uniformity in the field. In the overall balance of cardiovascular care, it dramatically reduces costs and unnecessary other tests or procedures, and improves outcomes.
